# Public Health Learning – Purposeful, Progressive, Global by Design

**DOI:** 10.3389/fpubh.2014.00230

**Published:** 2014-11-27

**Authors:** Susan Albertine

**Affiliations:** ^1^Association of American Colleges & Universities, Washington, DC, USA

**Keywords:** public health, public health learning, public health education, programs, undergraduates

Over the past decade, we have witnessed expansive growth in numbers and diversity of undergraduate public health programs. Majors, minors, and certificates are proliferating across all institutional types. While no complete census of programs exists, a number of studies have noted the growth (1, 2) [see also the special issue of *Am J Prev Med* (2008) 35(3), on undergraduate public health education]. The popular press has picked up the story (3). Community colleges are now developing programs (4). A key indicator: CEPH is now accrediting “standalone” baccalaureate programs. This growth phase builds on foundations laid earlier by a handful of schools, colleges, and programs, and the work of thought leaders such as Lilienfeld and Fraser (5). It was accelerated by the Institute of Medicine’s publication *Who Will Keep the Public Healthy* (2003), with its call for an educated citizenry: “all undergraduates should have access to education in public health” (144). Heeding that call, the Educated Citizen, and Public Health^1^ initiative has contributed in many ways to growth, in partnership with lead organizations – the Association for Prevention Teaching and Research (APTR), the Council of College of Arts and Sciences (CCAS), the Association of Schools and Programs of Public Health (ASPPH), and the Association of American Colleges and Universities (AAC&U). The recent merger of schools of public health and programs of public health, now joined in ASPPH, and growth in numbers of graduate programs overall may support further development of undergraduate public health programs.

The growth phase of public health education – a curricular movement at the undergraduate level – is a good thing. It has drawn the talents, energies, and passions of a wide array of academics, clinicians, and field practitioners. It has attracted such people as myself, a professor of American literature and English composition. Many of us see potential in the capacity of public health studies to embrace the full array of the liberal arts and general education, and offer a source of renewal and vitality to disciplines and fields under pressure in the twenty-first century. Many of us hope to see a more diverse population of students moving toward health professions through their introduction to public health. Because public health programs can offer highly applied and practical venues for learning in mathematics and the sciences, they may serve to open STEM fields to aspiring underrepresented minority students and start these students on pathways to the health professions (6). We see potential in the social and civic benefits that broader and more diverse access to education in public health can provide. I would venture to guess that few of us think anymore that the word *all* in the phrase “all undergraduates” was a typo in the IOM statement.

This growth phase has followed a predictable pattern. First, a flurry of interest. Then rapid activity in schools with accredited graduate programs. A few minors organized and then majors. Discovery of programs already in the field, including some in liberal arts colleges – more than had been collectively known. Then the headlong dash to build new programs. It has been a heady and exhilarating experience to witness so much energy, hope, and achievement.

The rapid growth of programs is unquestionably a good thing, but it does bring risks. One conjures up frontier images, complete with round-ups of perceived cash cows and feed lots for graduate education or the workforce. Think of the excitement and the folly of a land rush. Some of the lawlessness of frontier conditions certainly obtains. My readers will have heard the following questions: What, exactly, are all those undergraduates studying and learning? Where, after all, will all those hundreds and thousands of undergraduates go? What will they do? Who is preparing faculty to teach them? Who will hire them? We all hear these questions. As advocates, we quickly emphasize the bigger picture and greater good, the value of the outcomes to global health and wellbeing. We can point to examples and exemplars of success that institutions are achieving as they address these questions (7). The innovation, in other words, more than offsets the risks. The field is building capacity by growing the numbers of educated citizens and future practitioners who can act to improve public and global health in a profoundly challenging era. Yet questions associated with rapid growth are nonetheless important to consider and address.

Invoking images of risk, I mean to provoke thought. Someone needs to say – many of us need to say – that the field needs to behave in a far more intentional way than it is currently doing. I mean that educators on campuses need to be far more intentional, first, in developing programs designed appropriately for undergraduate learning. I also mean that educators must be more collaborative in a tough-minded, evidence-based way about the progression of student learning from high school and the beginning of college through entry into graduate programs or the workforce. Finally, I am calling for an intentional approach to learning from the associate through the baccalaureate to the graduate level that is globally attuned – and I do not mean simply “global” in content. We possess the tools and means, the principles, and philosophy for doing all of this work (8, 9). We have an opportunity to do what better-established disciplines and fields struggle to do. As we build, we carry responsibility to work wisely and well. If we believe so much depends on the lifelong successes of our students – beyond our individual courses and institutional programs – then we need to take time to be purposeful. This is manifestly a challenge to leadership at the academic and administrative levels, calling for a serious investment of resources as well as vigorous advocacy.

First, may I say with respect, as a critical friend and fellow public health educator – an undergraduate program is NOT a mini-MPH. All too often, we are seeing evidence of master’s level expectations set without due reflection in undergraduate programs. Why this should be happening is not hard to guess. Too few program leaders have had the time to think about curricular scaffolding, using tools to help them design purposeful learning that is appropriate for undergraduates. Because of the shortage of faculty, programs hire instructors who have domain knowledge and experience in public health but scant acquaintance with undergraduates. I have seen assignments developed for beginning baccalaureate students that would stretch the mind of entry-level master’s students. Beginning undergraduates struggle to identify scholarly articles or to draw distinctions between scholarship and popular media – or to read Wikipedia with a critical eye. A research assignment with a literature review pitched at the master’s level will defeat these students. If you observe this problem in your program, you should gather your faculty for an assignment exchange and a curricular design session. I have yet to see a group of faculty fail to identify the issues or to devise solutions. In fact, people typically enjoy this kind of collective work and sense that it needs to be done.

Resources are readily available. In my workshops, I use the Undergraduate Public Health Learning Outcomes and the Critical Component Elements^2^^,^^3^. Many of us know these frameworks but have not taken the time to use them with colleagues. A group together around the table with these materials in their hands will jump start collaboration. You have to take the step from theory to practice. Such interdisciplinary frameworks as these should likewise be available to students and faculty in practice settings.

If you also introduce one or two of the AAC&U VALUE rubrics^4^, you will discover a helpful synergy. The VALUE rubrics can help you to set expectations for learning as students move from novice stages in the domain of public health toward more independent expertise. If, for example, you want to think about the way students learn to carry out independent research, you can use the integrative learning or critical thinking VALUE rubrics. Developed by teams of faculty from many disciplines and fields, the rubrics describe learning as a sequence of performances that are generally appropriate and typical at the undergraduate level. Each rubric addresses a learning outcome that appears in the well-known and widely respected AAC&U framework of Essential Learning Outcomes (ELOs)^5^. Because the Undergraduate Public Health Learning Outcomes were themselves intentionally aligned with the ELOs, the crosswalk is far easier to make than you might imagine. For example, the critical thinking VALUE rubric offers a learning progression on the use of evidence. The progression begins with a “benchmark” assumption that undergraduates typically need to learn how to select and use information to investigate a point of view or conclusion. Undergraduates often begin by taking information from sources without any interpretation or evaluation, assuming the authority of all sources as given. By the time they graduate, we hope, they are able to take information from sources with sufficient discernment to develop a comprehensive analysis or synthesis, and to question the viewpoints of experts. The rubric suggests how students typically develop the knowledge and experience to undertake a research task. All 16 rubrics are readily applied within the content domain and methodologies of public health. VALUE rubrics are open-access tools that will make your program more coherent and purposeful, if you make the time to use them. You can also apply these rubrics to help your program increase the equity-minded and evidence-based practices that are highly effective with first-generation and multicultural students^6^.

Second, I have found it helpful to remind myself periodically that my students have come from somewhere and are going somewhere else. Teaching across generations can be challenging as students change and one’s experience builds. If you have yet to look at the Degree Qualifications Profile (DQP), you may be surprised how helpful it can be^7^. Developed by the Lumina Foundation, with leadership including AAC&U, and field tested in hundreds of colleges and universities since 2011, the DQP provides a profile of learning appropriate to the associate, baccalaureate, and master’s levels. It is aligned with the AAC&U ELOs and adds a new dimension to the ELOs and the VALUE rubrics. It actually profiles the degree as a whole. The DQP offers learning statements in five areas appropriate to both general education and major or specialized degree programs – as part of the overall progression of learning. The five are (1) specialized knowledge, (2) broad, integrative knowledge, (3) intellectual skills, (4) applied and collaborative learning, and (5) civic and global learning. For example, in the area of learning titled specialized knowledge – pertaining to the major program or field – the profile offers a spectrum of 10 learning statements. This continuum of statements begins with what students should be expected to achieve at the associate level and progresses through the master’s level. The first of these statements, at the associate level (or the undergraduate lower division): “Describes the scope of the field of study, its core theories and practices, using field-related terminology, and offers a similar explication of at least one related field.” At the bachelor’s level in specialized learning: “Defines and explains the structure, styles, and practices of the field of study using its tools, technologies, methods, and specialized terms.” At the master’s level, “Elucidates the major theories, research methods, and approaches to inquiry and schools of practice in the field of study, articulates theirs sources, and illustrates both their applications and their relationships to allied fields of study.” There are no bright lines of demarcation between the degree levels, but one observes how people are likely to grow as they learn. Even this brief extract should suggest how the DQP may be readily applied within learning domains of public health.

Third, as the DQP also suggests, global learning is not merely a content domain. It is a hugely meaningful educational context. As globalization reshapes education worldwide, we in the United States would be well advised to learn about degree frameworks that are developing in other countries, as for example in the European Union through the Bologna Process. The DQP invites us to address learning in global context. Within the field of public health, this invitation should be particularly resonant. As we think about purposeful and progressive learning for undergraduates, we should think with equal discernment about global health and global learning. ASPPH rightly makes the case that public health is global health^8^. We ought to think about educational frameworks that are globally attuned as a way to reach the IOM goal. Further, the public health community can and should promote efforts to align undergraduate public health programs with community-based global health needs, particularly for underserved populations.

Since 2009, I have been the steward of the Educated Citizen and Public Health (ECPH) listserv [list.aacu.org/mailman/listinfo/ecph]. The list is dedicated to undergraduate liberal education in public health. Every day, using Google Alerts, I scan the top hits in public health, looking for program items to share with ECPH. The experience has erased any distinction in my mind between global and public health. The search engine makes no such distinction, and neither should we. I and we should stop thinking about U.S. students and our programs in isolation. In and of itself this is a lesson in epidemiology. Right now Ebola dominates public health news. This is the world our students will inherit. We owe it to them to make their education in public health as purposeful, progressive, and global as we possibly can. We have the tools in our hands – and we bear a tremendous responsibility to use them well.

## Conflict of Interest Statement

The author declares that the research was conducted in the absence of any commercial or financial relationships that could be construed as a potential conflict of interest.

## Footnotes

^1^http://www.aacu.org/public_health/index.cfm

^2^http://www.aspph.org/educate/models/undergraduate-learning-outcomes/

^3^http://www.aspph.org/educate/models/undergraduate-baccalaureate-cce-report/

^4^http://www.aacu.org/value/index.cfm

^5^http://www.aacu.org/leap/vision.cfm

^6^http://www.aacu.org/sites/default/files/files/assessinghips/AssessingHIPS_TGGrantReport.pdf

^7^http://degreeprofile.org/download-the-dqp/

^8^http://www.aspph.org/discover/
